# Editorial: Immune tolerance and human malaria

**DOI:** 10.3389/fimmu.2024.1450480

**Published:** 2024-07-04

**Authors:** Katherine Rose Dobbs, Prasanna Jagannathan, Celia Dechavanne

**Affiliations:** ^1^ Center for Global Health and Diseases, Case Western Reserve University, Cleveland, OH, United States; ^2^ Division of Pediatric Infectious Diseases, University Hospitals Rainbow Babies and Children’s Hospital, Cleveland, OH, United States; ^3^ Division of Infectious Diseases and Geographic Medicine, Department of Medicine, Stanford University, Stanford, CA, United States; ^4^ Université de Paris, Institut de Recherche pour le Développement (IRD), Mère et enfant en milieu tropical (MERIT), Paris, France

**Keywords:** immune tolerance, human malaria, malaria in pregnancy, primed immunity, fetal immunity, disease tolerance

Exposure to malaria parasites induces immune modulations that affect susceptibility to malaria and other pathogens. Development of immunity to malaria results in protection against severe disease, though sterile immunity is typically not achieved (1, 2). Clinical immunity to malaria comprises both anti-parasite mechanisms that mediate parasite clearance and immunoregulatory mechanisms that limit immunopathology (tolerance). The mechanisms underlying tolerance to blood-stage infection are not well defined. Recent studies highlight the importance of immunoregulatory functions of cells such as monocytes (3), γδ T cells (4), and IL-10-producing T cells (5) in limiting immunopathology, though additional mechanistic studies are needed to understand the protective vs. pathogenic role these cells play in malaria.

The aim of this Research Topic was to provide further insights into the role of malaria-induced immune tolerance in protection against malaria. Mechanisms by which *Plasmodium* infections may lead to tolerized host responses, including in malaria in pregnancy, severe malaria in children, and *vivax* malaria, the impact of malaria exposures on malaria vaccine immunogenicity and potential strategies to overcome immune tolerance to improve vaccine efficacy were addressed in this Research Topic.

Changes in metabolism may underlie malaria-induced immune tolerance. Vandermosten et al. studied glucocorticoid signaling in children from Cameroon, comparing healthy children to those with uncomplicated or severe malaria. They found that plasma levels of glucagon and cortisol were elevated in children with clinical malaria. Stimulation of peripheral blood mononuclear cells with glucocorticoids *ex vivo* showed decreased glucocorticoid-induced gene expression and levels of cAMP in children with clinical malaria. These findings suggest that decreased leukocyte responsiveness to glucocorticoids may contribute to disease severity.

Diniz et al. evaluated the role of adenosine signaling in modulation of monocyte responses in patients with *P. vivax* infection. During a stress response, adenosine triphosphate (ATP) is released into the extracellular environment and delivers a danger signal upon binding to P2 purinergic receptors on innate immune cells. Ectonucleotidases tightly regulate extracellular ATP concentration by hydrolyzing ATP to adenosine. Adenosine binding to P1 purinergic receptors inhibits pro-inflammatory cytokine release and induces production of IL-10. In this article, the authors show that monocyte subsets from individuals with *P. vivax* infection display increased expression of ectonucleotidases CD39 and CD73 as well as increased expression of P1 purinergic receptors. Stimulation with adenosine decreased monocyte production of TNF, which was partially abolished by blocking the A_2a_ P1 purinergic receptor. The results suggest that adenosine may play an important role in modulating the inflammatory response during malaria.

Dechavanne et al. assessed the association between maternal malaria infection at delivery and the immune response in children aged 18 to 24 months. The authors found that intermediate and non-classical monocytes were less frequent in infant peripheral blood when they were born to mothers with placental malaria. They also found that those monocytes expressed higher levels of the LILRB2 inhibitory receptor compared to monocytes of infants to mothers without placental malaria. The long-lasting effect of maternal malaria infection on child monocytes raised the question about potential long-term functional impact on monocytes and children protection from infections.

Vianou et al. studied the phagocytic capability of subsets of monocytes in cerebral malaria. They found that non-classical monocytes were less frequent in peripheral blood, suggesting their recruitment to sites of inflammation. All three monocytes subsets were less capable of non-opsonizing phagocytosis and only the non-classical monocytes were less capable of opsonizing phagocytosis during cerebral malaria. The authors also found that LILRB2 expression increased in monocytes from children with cerebral malaria, along with other key inhibitory receptors, and expression of inhibitory receptors inversely correlated with the non-opsonic phagocytosis capability of those monocytes.

Intriguingly, some similarities have been found in monocytes from children born to mothers with placental malaria and from children with uncomplicated, severe or cerebral malaria. Phagocytic dysfunction of monocytes was also found in Dobbs et al. in children with uncomplicated malaria (6). Taken together, these studies suggest parasite strategies to modulate monocyte functions that possibly involve the engagement of inhibitory receptors, glucocorticoids and adenosine signaling. In Dechavanne et al. exposure to malaria in pregnancy was associated with long-term modulation of child monocytes at 18 months of age, with possible effects on child susceptibility to malaria or other infections.

Frimpong et al., investigated the TCR beta repertoire during malaria in children. The authors found a limited repertoire with a shorter CDR3 length in uncomplicated and severe cases compare to asymptomatic cases. They also found a much lower frequency of TCR with shared sequences between many individuals in severe *vs* asymptomatic cases. Taken together, these observations suggest a selection of TCR inducing immune response capable of controlling the symptoms at the moment of blood draw. In line with the poorly diversified TCR beta repertoire in severe cases, the authors previously observed a higher frequency of Treg and higher levels of inhibitory and senescent markers on T cells in children with clinical malaria (7).

Malaria-induced immune tolerance has important implications for malaria vaccine efficacy. Tiono et al. performed a secondary investigation using data from a clinical trial of the BK-SE36 vaccine, which targets the erythrocytic stage of *P. falciparum*. They found that individuals with concomitant infection with *P. falciparum* at the time of vaccination had decreased immunogenicity compared to uninfected individuals.

Duszenko et al. investigated a potential strategy to enhance immunogenicity of whole sporozoite (SPZ) vaccines. In endemic areas, suboptimal SPZ vaccine efficacy may be related to immune tolerance induced by previous malaria exposures. In this article, the authors demonstrate a proof-of-concept for enhancing SPZ immunogenicity, whereby the SPZ surface is chemically augmented with a TLR7 agonist-based adjuvant. The chemically augmented SPZ vaccine induced higher levels of IL-6 when macrophages were stimulated *in vitro* and increased SPZ-specific interferon (IFN)-γ production by NK cells, CD4^+^ T cells, and CD8^+^ T cells in immunized mice.

Taken together, these two last studies suggest that parasite infection modulates immune responses to vaccination and that a proper stimulation can restore innate and adaptive immune functions. Further studies that will define the role of immune tolerance in host protection against malaria may aid in improving vaccines.

## Author contributions

KD: Writing – original draft, Writing – review & editing. PJ: Writing – review & editing. CD: Writing – original draft, Writing – review & editing.
